# Fluorescence-based monitoring of ribosome assembly landscapes

**DOI:** 10.1186/s12867-015-0031-y

**Published:** 2015-02-25

**Authors:** Rainer Nikolay, Renate Schloemer, Silke Mueller, Elke Deuerling

**Affiliations:** Molecular Microbiology, University of Konstanz, Constance, 78457 Germany; Screening Center Konstanz, University of Konstanz, Constance, 78457 Germany; Current address: Institute of Medical Physics and Biophysics, Charité-Universitaetsmedizin Berlin, Berlin, 10117 Germany

**Keywords:** Ribosome assembly, Ribosome biogenesis, Fluorescent proteins, Antimicrobials, Knock out, λ-red recombineering, High throughput screening

## Abstract

**Background:**

Ribosomes and functional complexes of them have been analyzed at the atomic level. Far less is known about the dynamic assembly and degradation events that define the half-life of ribosomes and guarantee their quality control.

**Results:**

We developed a system that allows visualization of intact ribosomal subunits and assembly intermediates (i.e. assembly landscapes) by convenient fluorescence-based analysis. To this end, we labeled the early assembly ribosomal proteins L1 and S15 with the fluorescent proteins mAzami green and mCherry, respectively, using chromosomal gene insertion. The reporter strain harbors fluorescently labeled ribosomal subunits that operate wild type-like, as shown by biochemical and growth assays. Using genetic and chemical perturbations by depleting genes encoding the ribosomal proteins L3 and S17, respectively, or using ribosome-targeting antibiotics, we provoked ribosomal subunit assembly defects. These defects were readily identified by fluorometric analysis after sucrose density centrifugation in unprecedented resolution.

**Conclusion:**

This strategy is useful to monitor and characterize subunit specific assembly defects caused by ribosome-targeting drugs that are currently used and to characterize new molecules that affect ribosome assembly and thereby constitute new classes of antibacterial agents.

**Electronic supplementary material:**

The online version of this article (doi:10.1186/s12867-015-0031-y) contains supplementary material, which is available to authorized users.

## Background

The bacterial 70S ribosome is formed by a small 30S- and a large 50S subunit. While the small subunit consists of one 16S ribosomal RNA (rRNA) and 21 ribosomal proteins (r-proteins), the large subunit contains two rRNAs (23S and 5S rRNA) and 33 r-proteins [1]. Reconstitution of intact ribosomal subunits in the test tube is possible using components derived from purified ribosomes, but requires non-physiological conditions, such as high Mg^2+^ concentration and incubation temperatures of up to 50°C [2,3]. *In vivo*, this process critically depends on biogenesis factors, which are proteins that process, modify and chaperone rRNA or r-proteins [4,5]. Ribosome assembly is characterized by a highly coordinated sequence of events consisting of rRNA synthesis and r-protein uptake. Since assembly takes place co-transcriptionally (i.e. during rRNA synthesis) there is a hierarchical order of binding events with early- and late assembly r-proteins [5,6]. Each r-protein gene is present in a single copy per genome, whereas rRNAs are encoded by multiple *rrn* operons (seven ones in *E. coli*). One 16S, 23S and 5S rRNA (and several tRNAs) are contained in one primary transcript that is processed by site specific RNases [4]. Due to this genetic organization ribosomal subunits are consequently produced in equal stoichiometric amounts. Furthermore, synthesis of rRNA and r-proteins are synchronized [7] with the consequence that free cytosolic pools of r-proteins are close to zero under optimal conditions [8-10]. In addition, ribosome assembly is a fast process taking place within a couple of minutes at 37°C [11]. It follows that r-proteins upon synthesis are rapidly taken up by nascent ribosomal subunits. If selected r-proteins were fluorescence labeled, it further follows that the fluorescences signal would represent subunit precursors and whole subunits rather than free cytosolic pools of r-proteins.

Treatment of cells with chemical agents or occurrence of mutations (e.g. affecting genes encoding r-proteins or biogenesis factors) can lead to ribosome assembly defects [12]. One possible fate of defective assembly intermediates is a selective clearance by RNase based control mechanisms [13,14]. Alternatively, assembly intermediates can accumulate and possibly mature into intact subunits, as soon as the source of defect is eliminated [5,12,15-18].

Analyses of protein and RNA content of ribosomal assembly intermediates are possible using quantitative mass spectrometry approaches and cryo-electron microscopy [19-25]. Analyses of sucrose gradient fractions by agarose and two-dimensional gel electrophoresis with subsequent quantitation of rRNA and r-proteins are established methods but time-consuming and insensitive to small differences in quantity and quality.

Therefore, we set out to establish a convenient fluorescence-based method to assess amounts and assembly-states of ribosomal particles.

In our previous approach [26] late assembly r-proteins were labeled with fluorescent proteins (FPs) to monitor and compare the intact portions of both ribosomal subunits. In this study intact ribosomal subunits and assembly intermediates of all maturation states are detectable by labeling early assembly r-proteins. A reporter strain harboring the fusion proteins L1-mAzami green (a coral-derived monomeric green fluorescent protein [27], hereafter mAzami) and S15-mCherry was constructed and exhibited normal growth, indicating an intact translation apparatus. We used synthetic gene knock out of *rplC* (encoding L3) and *rpsQ* (encoding S17), respectively, or ribosome directed antibiotics to induce subunit assembly defects. A_254_ and fluorescence analysis of sucrose gradient centrifugates allowed *in vitro* analysis of ribosomal subunits and all of their assembly intermediates in unprecedented resolution.

## Results

### Rationale

In order to generate a reporter strain suitable for monitoring ribosome assembly landscapes, we selected ribosomal protein candidates from each subunit according to the following criteria [28,29]: The candidates should be i) distant from functional sites, ii) accessible to C-terminal tagging with fluorescent proteins, iii) early assembly proteins [10] and iv) subject of feedback regulation. The ribosomal proteins S15 and L1 fulfill all these criteria: Their surface exposed C-termini (Additional file 1A) allow convenient tagging (with mCherry and mAzami). Although these proteins are not essential [30] deletion strains have exaggerated generation times at 37°C. In addition, absence of S15 results in severe cold sensitivity [31] and ribosomes lacking L1 show 50% reduced translation activity *in vitro* [32]. Therefore, growth would be severely hampered if the fusion proteins do not fully complement the wild type protein’s function. According to *in vivo* ribosome assembly maps (Additional file 2), both are early assembly proteins and consequently present in ribosomal particles of each state of maturation. Finally, feedback regulation by autogenous control [33,34] ensures that they are not produced in excess.

### Phenotypic and biochemical characterization of the engineered strains

To generate *E. coli* strains harboring modified genes coding for S15-mCherry (MCr*) and L1-mAzami (MCg*) fusion proteins (Additional file 1B), we used the technique of lambda red recombineering [35,36]. The final reporter strain MCrg* producing both S15-mCherry and L1-mAzami fusion proteins was constructed, using P1 phage transduction.

**Figure 1:**
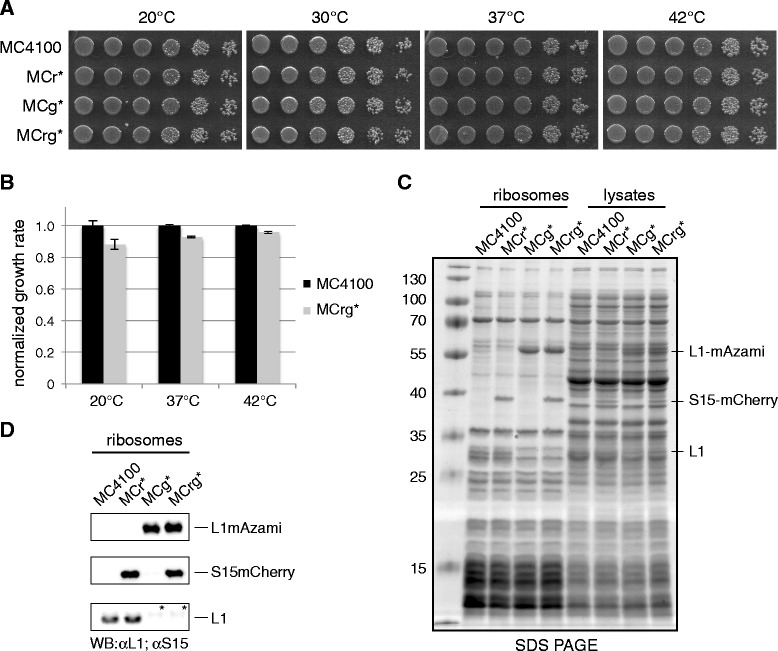
**Physiological and biochemical characterization. (A)** Growth comparison on solid medium: Cells of the indicated strains were spotted onto LB agar in a serial dilution and incubated at the given temperatures. **(B)** Cells as indicated were grown at 20, 37 and 42°C to stationary phase. Growth rates were calculated and normalized values are given for each strain at each incubation temperature. Data were obtained from three independent experiments. Ribosomes from the indicated strains were isolated by sucrose cushion centrifugation and subjected to SDS-PAGE **(C)** and western blot analysis **(D)**. For immunodetection, S15 and L1 specific antisera were used. Note that S15 wild type protein was not resolved by SDS-PAGE and immunoblotting. Asterisks denote unspecific protein bands.

To exclude that tagging of r-proteins with FPs interferes with regular cell functions and growth, we analyzed reporter strains in more detail. Spot tests revealed that growth of the genetically engineered strains did not differ from that of the wild type strain at various temperatures (Figure 1A). To analyze possible growth differences more precisely, all strains were grown to stationary phase at different temperatures and their growth rates were calculated (Figures 1B). It turned out that the growth rate of MCrg* at 37°C was 5-10% less than the wild type strain.

Next, the protein content of MCr*, MCg* and MCrg*-derived ribosomes was analyzed by SDS-PAGE and immunoblotting (Figure 1C, D). While MCr* and MCg* ribosomes contained one fusion protein (migrating at 37 and 57 kDa, respectively), two fusion proteins were observed in MCrg* ribosomes.

Collectively, the data indicate that growth behavior and functional competence of the ribosomes of MCrg* are similar to those of the parental strain.

### Generation of ribosome subunit specific assembly defects and *in vitro* analysis

To induce assembly defects in the small or the large ribosomal subunit, conditional gene knock outs of *rpsQ* (encoding S17) and *rplC* (encoding L3), respectively, were generated in the reporter strain background (Additional file 1A). It has been shown previously that defects in each of these genes caused ribosome assembly defects that were supposed to be subunit specific [26,37,38].

The resulting strains (MCrg*ΔsQ and MCrg*ΔlC) carried plasmids containing wild type copies of the genes deleted from the chromosome under control of an IPTG inducible promoter. The withdrawal of IPTG in liquid cultures should result in impaired growth and in subunit specific assembly defects [39] as soon as the number of intact ribosomes becomes limiting.

**Figure 2:**
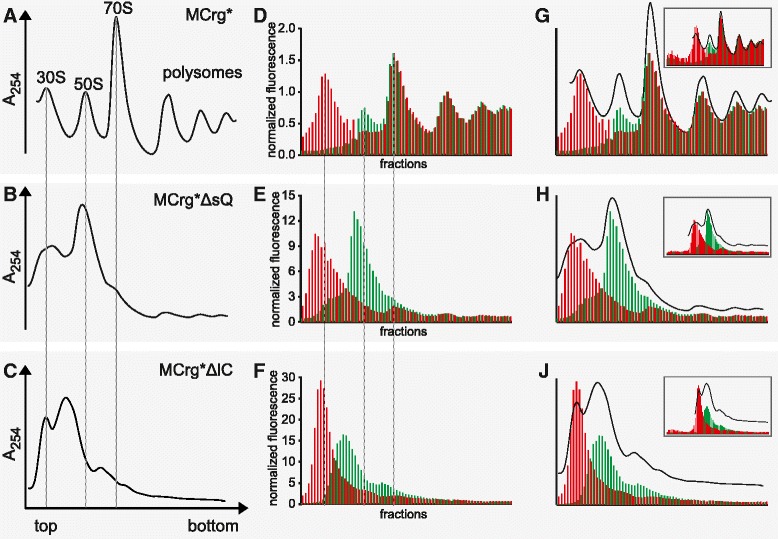
**Polysome analysis and fluorescence detection of sucrose fractions.** Cells were grown in M9 medium at 37°C to OD_600_ = 0.4 and harvested. Lysates were subjected to sucrose gradient centrifugation. Centrifugates were analyzed by A_254_ detection and fractionated. Polysome profiles derived from: **(A)** MCrg*, **(B)** MCrg*ΔsQ, **(C)** MCrg*ΔlC. Sucrose gradient fractions of samples **A-C** were analyzed for mAzami- and mCherry specific fluorescence and normalized results are given in bar charts for: **(D)** MCrg* **(E)** MCrg*ΔsQ **(F)** MCrg*ΔlC. Superposition of A_254_ profiles and corresponding fluorescence bar charts: **(G)** MCrg*, **(H)** MCrg*ΔsQ, **(J)** MCrg*ΔlC. The inserts show fluorescence analysis of all available fractions from each sucrose gradient run. Red bars: normalized mCherry fluorescence; Green bars: normalized mAzami fluorescence. Fluorescence was normalized to the first polysome peak (“disome”) where subunits are supposed to be present in 1:1 ratio. Experiments were done in duplicates, representative profiles are shown.

To this end, we grew MCrg*, MCrg*ΔsQ and -ΔlC cells in the absence of IPTG to mid-logarithmic phase and examined the ribosomes by sucrose gradient ultracentrifugation and polysome profile analysis (Figures 2A-C). MCrg* ribosomes showed the expected pattern consisting of 30S-, 50S-, 70S-, and polysome peaks (Figure 2A), whereas depletion of *rpsQ* (Figure 2B) led to dramatically reduced amounts of 70S ribosomes and polysomes, increased amounts of 50S subunits and a broad peak of particles in the region of the 30S subunits. Likewise, depletion of *rplC* reduced the amount of 70S ribosomes and led to a defined peak of 30S subunits and a large and broad peak of particles migrating between mature 50S and 30S subunits (Figure 2C). This was expected because both absence of *rpsQ* and *rplC* should result in defective small and large ribosomal subunits, respectively. Consequently, the reduced number of functional subunits limited the amount of monosomes and polysomes.

Fluorometric analysis of the sucrose fractions provided fluorescence profiles of MCrg*, MCrg*ΔsQ and -ΔlC derived ribosomes (Figures 2D-F). Comparing A_254_ and fluorescence profiles of MCrg* ribosomes (Figure 2G) revealed reasonable coincidence of the individual peaks. For completeness the entire profile, also including early low molecular weight fractions, is depicted as insert (Figure 2G, insert).

When analyzing A_254_ and fluorescence profiles of MCrg*∆sQ ribosomes by overlay (Figure 2H) several aspects attracted attention: The largest peak of red fluorescence -representing the small subunit- was decreased in intensity relative to largest peak of green fluorescence -representing the large subunit- (in comparison with Figure 2G). Moreover, the red fluorescence peak was in addition left shifted due to absence of *rpsQ*. The largest peak of green fluorescence was slightly left shifted and showed a shoulder at the lower left side overlapping with the red peak, indicating defective large ribosomal subunits. This indicates that a selective assembly defect of the small subunit is also associated with an accumulation of 50S assembly intermediates. Finally, as shown in the insert there was no increased fluorescence in the low molecular weight fractions, indicating proper autogenous control of S15-mCherry and L1-mAzami.

Combined analysis of A_254_ and fluorescence profiles of MCrg*∆lC ribosomes (Figure 2J) revealed a decrease in the green fluorescence peak relative to the red fluorescence peak, in comparison with Figure 2H. In addition, the green fluorescence peak was clearly left shifted, due to assembly defects in the absence of *rplC*. In the A_254_ profile the peak of the large subunit appeared higher than the peak of the small subunit. This is presumably the case because peaks of both subunits are overlapping each other (Figure 2F), thereby producing a dominant broad peak in the region of the large subunit. Investigation of the low molecular weight fractions in the insert showed strict feedback regulation of S15-mCherry and L1-mAzami.

In summary, assembly defects of the small and large ribosomal subunit could be provoked and were readily detectable by fluorescence analysis of sucrose gradient centrifugates.

### *In vivo* analysis of subunit specific assembly defects

Next, we asked whether subunit assembly defects could be detected by fluorescence readout *in vivo* using MCrg*? Fluorescent labeling of the early assembly r-proteins L1 and S15 results in fluorescent subunits of all stages of maturation. Since both subunits are systemically produced in equal amounts, any shift in the fluorescence ratios is expected to be a consequence of subunit specific turnover (facilitated by ribonucleases and proteases). Increased turnover of the large subunit should reduce the amount of green fluorescence and consequently lower the normalized fluorescence emission ratio of mAzami/mCherry, while increased turnover of the small subunit in turn should increase the ratio.

**Figure 3:**
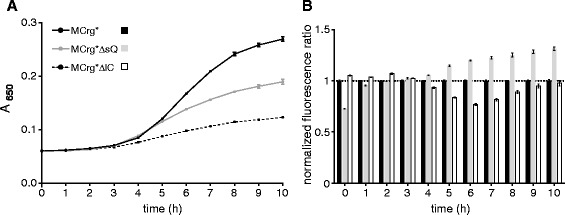
**Growth in 384-well pates and fully automated fluorescence analysis of reporter strains.** Aliquots of MCrg*, MCrg*ΔsQ and MCrg*ΔlC cultures were transferred into 384- well plates in quadruplicates. Cells were grown in M9 medium at 37°C for 10 hours. Measurements were made in one-hour intervals. **(A)** A_650_ values were determined and **(B)** mAzami and mCherry fluorescence emission were detected and ratios were calculated for MCrg*, MCrg*ΔsQ and MCrg*ΔlC. Fluorescence ratios of MCrg* were normalized to 1. Data points given in the growth curves and fluorescence ratios are mean values from four independent experiments; error bars show standard deviation.

MCrg*, MCrg*ΔsQ and MCrg*ΔlC cells were transferred to 384-well plates and incubated at 37°C for 10 hours in M9 medium. Fully automated sample handling was possible, using a robotic platform equipped with incubator, microplate reader and robotic arm. Both A_650_ values and fluorescence intensities were measured (Figure 3) in one-hour intervals. From the latter, normalized fluorescence ratios were calculated. While MCrg* grew unperturbed, MCrg*ΔsQ and MCrg*ΔlC cells showed impaired growth and reached lower cell densities after 10 hours (Figure 3A). The background corrected and normalized fluorescence ratios of MCrg*ΔlC reached a minimum of 0.8 after 6 hours, revealing a defect of the 50S assembly, whereas the ratios of MCrg*ΔsQ increased instead reaching a maximum of 1.3 after 9 hours indicating a defect in the 30S assembly (Figure 3B).

We conclude that depletion of *rplC* and *rpsQ*, respectively, causes severe assembly defects of ribosomal subunits. Moreover, the changes in fluorescence ratios suggest that there is turnover of defective subunits.

### Probing MCrg* with ribosome-targeting antibiotics

It has been shown that translation inhibitors such as chloramphenicol [16,17,40,41], erythromycin [16,17,42,43] and neomycin [18,44,45] cause directly or indirectly assembly defects of both ribosomal subunits. The mechanistic interpretation of the antibiotic mediated effects is controversial [15-18]. We tested all of the before mentioned antibiotics and included a fourth one (kanamycin) that, to our knowledge, was not investigated so far for its potential to cause ribosome assembly defects. Using MCrg*, we set out to clarify, whether treatment of cells with ribosome targeting antibiotics results in assembly defects of one or both of the ribosomal subunits. The fact that early assembly proteins are fluorescently labeled should allow detailed analysis of the assembly landscapes upon antibiotic treatment. In addition, cell based assays were used to elicit whether there are indications for subunit specific turnover.

**Figure 4:**
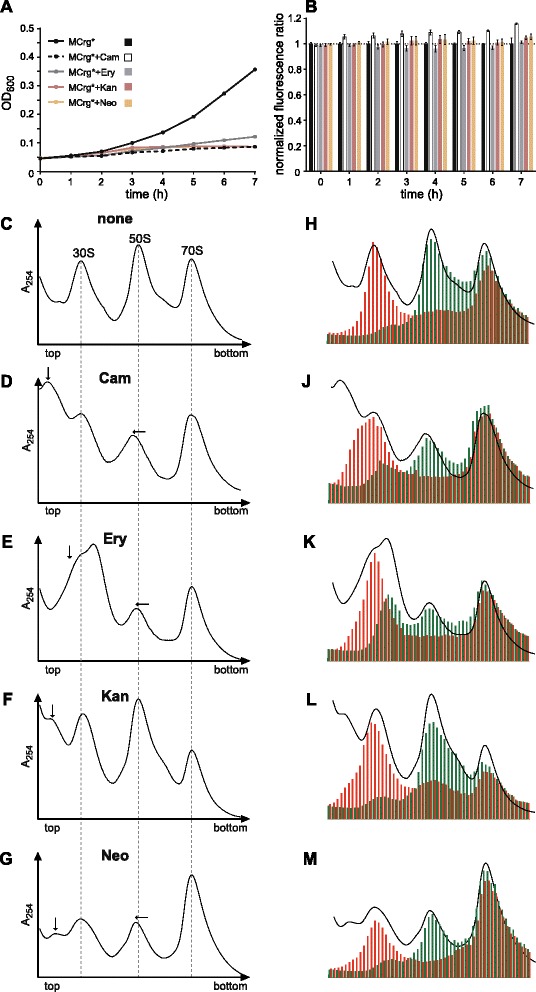
Testing MCrg* with inhibitors of translation. Cell-based assay: MCrg* cells were cultured in Erlenmeyer flasks at 25°C in M9 medium for 7 hours in the absence and presence of antibiotics, as indicated. Samples were taken every hour. **(A)** OD_600_ values were determined and **(B)** mAzami and mCherry fluorescence emission were detected and ratios were calculated. Fluorescence ratios of MCrg* were normalized to 1. Exemplary growth curves are given and fluorescence ratios are means from three independent experiments; error bars show standard deviation. **Analyses of isolated ribosomal particles**: Sucrose density gradient (10-25%) centrifugation profiles from **(C)** control cells with no antibiotic (none), **(D)** chloramphenicol (Cam), **(E)** erythromycin (Ery), **(F)** kanamycin (Kan) and **(G)** neomycin (Neo) treated cells. Sucrose gradient fractions from **(C)** to **(G)** were analyzed for fluorescence by a microplate reader. A_254_ profiles and fluorescence bar charts were superimposed for **(H)** control cells with no antibiotic (none), **(J)** chloramphenicol (Cam), **(K)** erythromycin (Ery), **(L)** kanamycin (Kan) and **(M)** neomycin (Neo) treated cells. Cells in presence and absence of antibiotics were cultured in LB medium at 25°C for 3 hours before subsequent polysome analysis. Left shifted peaks of the large subunit are indicated by horizontal arrows, abnormal portions of the small subunit by vertical arrows. Red bars: normalized mCherry fluorescence; Green bars: normalized mAzami fluorescence. Fluorescence was normalized to the first polysome peak (“disome”) where subunits are present in 1:1 ratio. Experiments were done in duplicates, representative profiles are shown.

MCrg* cells were grown in M9 medium for seven hours in the absence or presence of chloramphenicol (7 μg/ml), erythromycin (100 μg/ml), kanamycin (7 μg/ml) or neomycin (7 μg/ml). While each antibiotic led to impaired cell growth (Figure 4A), chloramphenicol caused strongest growth defects. Erythromycin, kanamycin and neomycin treatment did not show a significant change in fluorescence ratios (Figure 4B). Treatment with chloramphenicol, by contrast, led to an increased fluorescence ratio, with a maximum of about 1.20 after 7 hours. This suggests that treatment of cells with chloramphenicol might decrease the relative amounts of the small subunit.

This hypothesis was tested by analyzing ribosome profiles obtained from MCrg* cells that grew in the presence of the antibiotics or without (Figure 4C-G).

Figure 4C shows the A_254_ profile of ribosomes derived from non-treated cells. The 70S peak and the polysomes (not shown) were reduced in intensity since no chloramphenicol was added prior to harvesting. In our previous study, it turned out that addition of chloramphenicol (immediately before harvest) to antibiotic-treated cells caused extremely broadened 70S peaks. Ribosomes derived from chloramphenicol (D) or erythromycin (E) treated cells showed reduced and left shifted 50S peaks. Shifted peaks of the large subunit are indicated with horizontal arrows, additional peaks and shoulders left-lateral of the 30S peak are marked with vertical arrows. Chloramphenicol treatment caused an additional peak (left-lateral of 30S), while after erythromycin treatment the 30S peak had a shoulder on the left. Treatment with kanamycin had no apparent influence on the 50S peak but provoked an additional peak left-lateral of the 30S peak (F). Neomycin treatment caused a slight reduction and left shift of the 50S peak and an additional peak on the left of the 30S peak (G).

Overlay diagrams (combining A_254_ and fluorescence read outs) (Figure 4H-M) show that ribosome profiles from non-treated cells are congruent (Figure 4H). Profiles from chloramphenicol and in particular erythromycin treated cells revealed an extra portion of green fluorescence within the region of the 30S peak (Figure 4J-K). Ribosome profiles derived from kanamycin and neomycin treated cells possessed additional green fluorescent peaks of very weak intensity but at similar positions within the profile (Figure 4L-M). The particles within the green peaks migrating slower than that of the 50S subunits presumably represent defective assembly intermediates of the large subunit that are caused by all the antibiotics used in this study, but to different extent. For a more detailed analysis the green fluorescence profiles were aligned and the one derived from non-treated cells was compared with all the others obtained from antibiotic treated cells (Additional file 3), illustrating the presence of assembly intermediates of the large subunit. Comparison of the red fluorescent peaks (Figure 4H-M) indicates that treatment with all antibiotics produced a more or less pronounced shoulder on the left side. To analyze potential defects of the small subunit in more detail, the red fluorescence profiles derived from antibiotic treated cells were compared with the one obtained from non-treated cells (Additional file 4). It turned out that treatment with all four antibiotics caused distinct left-sided shoulders of the red fluorescence peak, indicating small subunit assembly defects. This is either due to accumulation of the 21S precursor of the 30S subunit, or assembly dead-ends of this subunit. A more thorough analysis, which includes quantitation of the 16S and 23S rRNA within the sucrose fractions is given in the supplementary material (Additional file 5), confirms all hypothesized assembly intermediates. Taken together, the administration of a representative collection of ribosome-targeting antibiotics caused assembly defects of both subunits in each case, but to different extents.

In summary, our analyses demonstrated that ribosome assembly defects are detectable by fluorometric analysis of fractions collected after sucrose gradient ultracentrifugation of ribosomal preparations. Assembly defects caused by gene depletions (of *rplC* and *rpsQ*) or by treatment of reporter cells with four different ribosome-targeting antibiotics revealed the presence of defective assembly intermediates of both ribosomal subunits.

## Discussion

Here, we report construction and characterization of the strain MCrg* harboring large and small ribosomal subunits labeled with mAzami and mCherry proteins, respectively. This strain has growth properties similar to the parental strain. We have provided evidence that the reporter strain reveals assembly defects of ribosomal subunits by fluorescence-based readouts of sucrose density centrifugates and should report differences in subunit specific turnover when subjected to fluorescence-based *in vivo* analysis.

In our previous study, we labeled the late assembly r-proteins L19 and S2 with FPs to identify and compare the portion of intact ribosomal subunits. A detailed characterization of physiological and biochemical properties of the reporter strain demonstrated that there were no substantial limitations of the translation apparatus resulting from labeling of two ribosomal proteins with two different FPs. When ribosome assembly had been perturbed genetically or chemically fluorescence-based read out allowed identification of these assembly defects [26].

In the current study, we labeled the early assembly r-proteins L1 and S15 with FPs. Again, we made sure that tagging of the r-proteins with FPs did not compromise cell physiology and ribosome composition, as confirmed by growth assays and SDS-PAGE analysis of purified ribosomes (Figure 1). In addition, we provided evidence that MCrg* allows for *in vitro* analysis of isolated ribosomes to monitor fully matured ribosomal subunits and their assembly intermediates (Figure 2). Moreover, MCrg* is a strong tool for cell-based assays. It allows fluorometric *in vivo* determination of the ratio between the two ribosomal subunits, including all assembly states. In untreated reporter cells this ratio is expected to be one, due to equal production of ribosomal subunits. A change in the fluorescence ratio is therefore interpreted as consequence of unequal turnover of ribosomal subunits.

### Generation of subunit specific assembly defects by gene depletion

Subunit specific ribosome assembly defects were induced by depletion of r-protein genes encoding the early assembly proteins S17 and L3, respectively (Figure 2). Depletion of *rpsQ* (encoding S17) caused an assembly defect of the small subunit (Figure 3E) and increased the fluorescence ratio by 30% (Figure 3B), indicating increased turnover of the small subunit. Depletion of *rplC* (encoding L3) caused an assembly defect of the large subunit (Figure 2F) and decreased the fluorescence ratio by 20% (Figure 3B), indicating increased turnover of the large subunit. It seems that assembly defects on one subunit disturb assembly of the other subunit. It is possible that assembly defects reduce the pool of active 70S ribosomes, thereby reducing levels of translation, which in turn induces in assembly defects of both subunits (also see below).

### Treatment of MCrg* with ribosome targeting antibiotics

Chloramphenicol and erythromycin target the large ribosomal subunit, while kanamycin and neomycin interact predominantly with the small ribosomal subunit. Chloramphenicol binds to the peptidyl transferase center (PTC) and inhibits fixation of the CCA-aminoacyl end of an aminoacyl tRNA at the A-site region of the PTC [46], while the macrolide erythromycin binds in the upper region of the ribosomal exit tunnel and hinders protein synthesis by blocking a full occupation of the exit tunnel [47]. Kanamycin and neomycin belong to the aminoglycoside and 2-deoxystreptamine aminoglycoside families of antibiotics, respectively. They both target the 30S subunit and interact with the internal loop 44 of the decoding center [48]. For neomycin an additional binding site within helix 69 of the 23S rRNA was reported [49,50]. Both antibiotics cause translational misreading by stabilizing and binding of near cognate tRNAs to the mRNA [48].

Antibiotics used in this study induce assembly defects in both ribosomal subunits (Figure 4). This raises the question, whether a perturbation of the translation apparatus always results in mutual assembly defects? According to previous studies, inhibition of translation by ribosome targeting antibiotics reduces protein biosynthesis and provokes overproduction of rRNA [16,51,52]. Consequently, there is a stoichiometric imbalance between rRNA and r-proteins, which promotes accumulation of defective precursor particles of both ribosomal subunits [5,16]. Siibak et al. provided evidence that the presence of sublethal concentrations of chloramphenicol or erythromycin resulted in reduced and unbalanced protein biosynthesis [16,17]. In particular ribosomal proteins were produced in amounts that correlated with their presence in assembly intermediates [17]. In that sense assembly defects reflect an indirect consequence of impaired translation.

In our study defective subunits were monitored by fluorescence-based *in vitro* analyses in great detail. Notably, the defects observed in subunit assembly upon treatment with antibiotics were most reveling (Figure 4, Additional files 3 and 4). Ribosome profiles clearly differed both in A_254_ and in fluorescence analysis. Detailed analyses of 30S subunit formation *in vitro* [53] and evidence from *in vivo* experiments [18] suggest that there are parallel routes for ribosomal subunit formation. Therefore, assembly intermediates described here (and in literature) may reflect a heterogeneous collection of similar sizes and shapes [5]. This could indicate that the used antibiotics have intrinsic properties that cause specific assembly defects with particles slightly differing in size and composition. Champney and coworkers have provided evidence, using different bacterial species and dozens of different antibiotics, that certain ribosome targeting drugs bind to ribosomal precursor particles and thereby presumably hinder further maturation to intact subunits [15,54].

Even though mechanistic concepts and causative explanations for ribosome assembly defects caused by current translation inhibitors differ, attempts to identify primary inhibitors of ribosome assembly are important and worthwhile for several reasons:

1. The availability of small molecules that selectively inhibit the assembly process of one subunit at a defined stage would clearly be highly appreciated by researchers in the ribosome field to obtain a more detailed understanding of subunit specific assembly *in vivo*.
2. Such compounds could be the basis for the development of drugs with a defined target leading to subunit specific assembly breakdown. Molecules can be envisioned that exclusively target either assembly of the large or the small ribosomal subunit.
3. If the latter was possible, these compounds could be combined in one preparation to achieve defined and sustained assembly defects of both ribosomal subunits simultaneously.

## Conclusions

The described here fluorescence-based assays allow for monitoring of ribosome assembly landscapes in hitherto unmatched resolution. Assembly intermediates can be precisely detected and unambiguously allocated to either the large or small ribosomal subunit. Rather than replacing quantitative mass spectrometry approaches, it is supposed to be a valuable method to select the optimal fractions for subsequent mass spectrometric or structural analyses. Therefore, we believe that MCrg* significantly enriches the tool kit for a more thorough investigation of ribosome assembly and characterization of new classes of antimicrobials. The methodology described here might also be applied to eukaryotic systems from yeast up to mammalian cells, using CRISPR Cas 9 based knock-in techniques [55-57] to screen for small molecule inhibitors of eukaryotic ribosome assembly. The high metabolic activity of tumor cells goes along with high rates of protein production, which is expected to make them more susceptible towards inhibition of the translation apparatus than normal cells [58]. Therefore, molecules found in such screenings could be the basis for the development of new anti-cancer drugs.

## Methods

### Media, buffers, antibodies and antibiotics

LB medium (5 g yeast extract, 10 g trypton, 5 g NaCl/ l); M9 medium (64 g Na_2_HPO_4_^.^7H_2_O, 15 g KH_2_PO_4_, 2.5 g NaCl, 5 g NH_4_Cl, 2 mM MgSO_4_, 0.1 mM CaCl_2_, 0.4% glucose/l); PBS (137 mM NaCl, 2.7 mM KCl, 10 mM Na_2_HPO_4_, 1.8 mM KH_2_PO_4_, pH 7.4); S15 and L1 specific antisera, raised in sheep were obtained from Dr. Nierhaus. Horseradish peroxidase (HRP)-conjugated rabbit anti-sheep (CodeNo: 313-035-003; LotNo: 106383) and donkey anti-rabbit (CodeNo: 711-035-152; LotNo: 103871) secondary antibodies were from Jackson ImmunoResearch. HRP-substrate: for detection a mixture of 1 ml solution A + 100 μl solution B + 1 μl solution C was freshly prepared (solution A: 0.1 mM TRIS (pH8.6), 25 mg Luminol, 100 ml distilled H_2_0; solution B: 11 mg *p*-hydroxycoumaric acid, in 10 ml DMSO; solution C: H_2_O_2_ (30%). Antibiotics were used in concentrations as indicated: Ampicillin (Applichem-A0839,0100), chloramphenicol (Sigma-C0378), erythromycin (Sigma-E6367), kanamycin (Roth-T832.4) and neomycin (Sigma-N1876).

### Plasmids and bacterial strains

*rpsQ* and *rplC* were amplified from genomic *E. coli* DNA using specific primers with *Sac*I and *Xba*I restriction sites, respectively. Digested inserts were ligated with an opened pTRC99a vector (*lacIq*, trc promoter, *bla*-gene for ampicillin resistance) [59] using *Sac*I/ *Xba*I restriction sites generating pTRC-*rplQ* and pTRC-*rplC*, respectively. Plasmids were brought into DH5α-Z1 by chemical transformation for amplification and were isolated using Qiagen mini-prep kit.

**MC4100** (F^−^*[araD139]*_*B/r*_*Δ(argF-lac)169 lambda*^*−*^*e14- flhD5301 Δ(fruK-yeiR)725 (fruA25) relA1 rpsL150(strR) rbsR22 Δ(fimB-fimE)632(::IS1) deoC1*); **DY330** (W3110 *Δ lacU169 gal490 λcI857 Δ (cro-bioA)*)[36], **DH5α-Z1** (F *endA1 hsdR17*(r_k_ m_k+_) *supE44 thi-1 recA1 gyrA relA1* Δ (*lacZYA-argF*)U169 *deoR* Ф80 *lacZ*Δ M15 *Lac*R *Tet*R and *Sp*^*r*^) [60]

### λ-red recombineering

Coding sequences of mAzami green [27] (hereafter mAzami) and mCherry (fused with kanamycin resistance cassettes (kanR) derived from plasmid pKD4 [35]) with flanking homologous regions (40–50 nucleotides) for 3’prime genomic insertion in frame with *rplA* and *rpsO*, respectively, were amplified using Phusion DNA-Polymerase. PCR products of the expected size were purified and brought into competent DY330 cells via electroporation. Successful genomic integration was verified by colony PCR and DNA-sequencing. Genetic modifications were transferred to strains of interest using P1-phage transduction. Resistance cassettes were eliminated by transforming strains of interest with pCP20 [61] encoding FLP-recombinase from *Saccharomyces cerevisiae*, which eliminates kanR flanked by FRT-sites. Strains were cured from pCP20 by incubation at 42°C for 15 hours. Gene deletions of *rpsQ* and *rplC* were achieved as described previously [26].

### Cell growth analysis

Growth on LB agar plates: Stationary *E. coli* cells were diluted in LB medium to an initial cell density of OD_600_ = 0.025. 1:5 serial dilutions were prepared and transferred to LB agar plates with a plating stamp. Agar plates were incubated at 20, 30, 37 and 42°C until visible single colonies had formed.

Growth in liquid media: Stationary *E. coli* cells as indicated (cultured in LB medium) were diluted in LB medium to an initial cell density of OD_600_ = 0.05 (for incubation at 20°C) or 0.025 (for incubation at 37 and 42°C). Alternatively, stationary pre-cultures of the indicated strains were washed and diluted in M9 medium to an initial cell density of OD_600_ = 0.05. Cell suspensions were cultured in baffled flasks in a water bath incubator with a shaking frequency of 200 rpm until stationary phase was reached or a maximum of 10 hours had passed. Cell density was determined using a photometer (Amersham ultrospec 3110 pro). Growth rates were calculated for periods of exponential growth.

### SDS-PAGE and immunoblot analysis of purified ribosomes

Proteins from cell lysates (20 μg total protein) and purified ribosomes (15 pmol) from the indicated strains were resolved by a 13% SDS gel. Proteins were stained using Coomassie brilliant blue 250G.

For immunoblot analysis proteins from cell lysates (3 μg total protein) and purified ribosomes (3 pmol) were resolved by a 13% SDS gel and blotted to nitrocellulose membranes, which were decorated with S15 (raised in rabbit) and L1 (raised in sheep) specific anti-sera (1:10000 and 1:15000 in TBS +3% milk powder). HRP-conjugated donkey anti-rabbit and rabbit anti-sheep secondary antibody (1:20000 and 1:10000 in TBS + 3% milk powder) were used in combination with HRP-substrate to allow immunodetection. Chemiluminescence was monitored using LAS 3000 imager (Fuji Film).

### Purification of ribosomes by sucrose cushion centrifugation

*E. coli* cells were cultured in LB medium at 37°C to cell densities as indicated, harvested by centrifugation and processed as described previously [26].

### Sucrose gradient centrifugation and ribosome analysis

Stationary pre-cultures of the individual strains were washed and diluted in M9 medium to OD_600_ = 0.05 and cultured at 37°C to cell densities as indicated. Chloramphenicol (250 μg/ml) was added 5 min before cells were harvested by centrifugation, flash-frozen, and stored at −80°C. Frozen cell pellets were resuspended in buffer IV (10 mM TRIS, 10 mM MgCl_2_, 100 mM NH_4_Cl, 250 μM chloramphenicol, pH7.5) and cells were lyzed using Fastprep-24. Cleared lysates (0.5 ml of a solution with A_260_ = 15 or 20) were loaded on 10-40% sucrose gradients (in buffer V: 10 mM TRIS, 10 mM MgCl_2_, 100 mM NH_4_Cl, 0.5 mM DTT, 1xTM Complete, pH7.5) and centrifuged in a Sorvall TH-641 rotor for 2:40 h at 41 krpm. A_254_ profiles of sucrose centrifugates were obtained using a Teledyne Isco gradient reader. Fractions of the sucrose gradient were collected in 96-well plates (5 drops per well) for further fluorometric analysis.

For testing MCrg* with antibiotics, cells were washed and diluted in M9 medium to OD_600_ = 0.1 and grown to OD_600_ = 0.15 before chloramphenicol (7 μg/ml), erythromycin (100 μg/ml), kanamycin (7 μg/ml) or neomycin (7 μg/ml) were added. Cells were cultured at 25°C. After 3 hours of incubation cells were harvested and processed as described above with two exceptions: No Chloramphenicol was added 5 min before harvesting and 10-25% sucrose gradients were used for separation of ribosomal particles.

### Agarose gel electrophoresis

20 μl of sucrose gradient fractions were mixed with 6xDNA sample buffer (Thermo Scientific) and loaded on a 1% agarose gel. The RNA content was separated at 150 V for 1 hour. The gel was stained in an ethidium bromide solution for 15 min and RNA bands were visualized at 302 nm on an UV-transilluminator (UVP) by optical inspection.

### Fluorometric analyses

Manual measurements using Fluorospectrometer (Jasco FP-6500): 1 ml aliquots of cell suspension of various strains were transferred to quartz cuvettes and mAzami- (excitation 480 nm/ emission 510 nm ± 5 nm band width) and mCherry specific fluorescence intensities (excitation 580 nm/ emission 610 nm ± 5 nm band width) were determined. Fluorescence ratios were calculated by dividing mAzami by mCherry fluorescence intensities. Ratios were normalized to the reporter strain (MCrg*) and plotted in bar charts.

For the fully automated in *vivo assay* a Freedom EVO® 200 robotic platform (Tecan) was used, as described before [26]. Briefly, stationary cells of various strains were washed and diluted in M9 medium to a cell density of OD_600_ = 0.05. 80 μl aliquots of each strain were transferred to a 384-well plate in quadruplicates. Cells were incubated for 10 hours at 37°C in a monitored incubator (MIO2™) with 8.5 Hz shaking frequency. The samples were analyzed in a microplate reader in one-hour intervals for mAzami- and mCherry specific fluorescence, using filter combinations 485/535 nm and 535/612 nm, respectively. Fluorescence ratios were calculated by dividing mAzami by mCherry fluorescence intensities. Background corrected ratios were normalized to the reporter strain (MCrg*) and plotted in bar charts. Cell densities were determined simultaneously by detecting the absorbance at 650 nm ± 5 nm (A_650_). Obtained values were plotted in a spread chart. Calculations and diagrams were made using Magellan 7 (Tecan) and Graph Pad Prism v6 (Graph Pad) software packages.

Sucrose fraction were analyzed using an Infinite F500 (Tecan) fluorescence microplate reader. Sucrose gradient fractions collected in 96-well plates (5 drops per well) were analyzed for mAzami- and mCherry specific fluorescence using filter combinations 485/535 nm and 535/612 nm, respectively. Fluorescence intensities were normalized to a fraction containing the first polysome (“disome”) peak, as indicated. In Figure 4 and Figures based on it, the polysome peaks are not shown. Nevertheless, in each case the first polysome peak was used for normalization.

## Additional files

Additional file 1:
**Overview constructed strains and 70S ribosome structure.** (A) Surface representation of a T. termophilus 70S ribosome crystal structure. The 16S rRNA is colored in light gray proteins of the small subunit in yellow. 23S and 5S rRNA are shown in dark gray, proteins of the large subunit in cyan. S15 is highlighted in red, L1 in green. Their surface exposed C-termini are shown in purple. The figure was generated with pymol, based on PDB files 4KCZ and 4KCY (29). (B) Given are the names of the constructed strains as used in this study (lab nomenclature in brackets), relevant genotype and fluorescent fusion proteins produced. pTRC-rpsQ, pTRC-rplC: complementation plasmids with copies of the chromosomally deleted genes. Endogenous genes are shown as gray arrows, genes encoding fluorescent proteins as colored boxes. Genes to be deleted were replaced by kanamycin resistance cassettes (KanR). mCherry gene and protein portions are shown in red, mAzami accordingly in green. Additional file 2:
***in vivo*** **30S and 50S ribosome assembly maps adapted from** **[** 10 **].** (A) 30S assembly map reflecting order and interdependency of r-protein interaction with the nascent small ribosomal subunit. Early interacting r-proteins are shaded in dark gray, late interacting ones in light gray. Boxed proteins are contained in the 21S precursor of the 30S subunit. S15 is circled in red. (B) 50S assembly map reflecting order and interdependency of r-protein interaction with the nascent large ribosomal subunit. Early interacting r-proteins are shaded in dark gray, late interacting ones in light gray. Proteins contained in the 32S or in the 43S precursor of the large ribosomal subunit are boxed or circled in black, respectively. L1 is circled in green. Additional file 3:
**Alignment of mAzami specific fluorescence intensities from samples analyzed in Figure 4.** mAzami fluorescence profiles from untreated cells in combination with mAzami profiles derived from (A) chloramphenicol (Cam), (B) erythromycin (Ery), (C) kanamycin (Kan) and (D) neomycin (Neo) treated cells. The diagrams show normalized mAzami fluorescence intensities from sucrose fractions derived from untreated cells (gray bars) in direct comparison with the ones from antibiotic treated cells (green bars). 70S peaks were used for normalization. Additional file 4:
**Alignment of mCherry specific fluorescence intensities from samples analyzed in Figure 4.** mCherry fluorescence profiles from untreated cells in combination with mCherry profiles derived from (A) chloramphenicol (Cam), (B) erythromycin (Ery), (C) kanamycin (Kan) and (D) neomycin (Neo) treated cells. The diagrams show normalized mCherry fluorescence intensities from sucrose fractions derived from untreated cells (gray bars) in direct comparison with the ones from antibiotic treated cells (red bars). 70S peaks were used for normalization. Additional file 5:
**A254 detection and fluorescence analysis of sucrose density gradient fractions.** Sucrose density gradient (10-25%) centrifugation profiles derived from (A) control cells with no antibiotic (none), (B) chloramphenicol (Cam), (C) erythromycin (Ery), (D) kanamycin (Kan) and (E) neomycin (Neo) treated cells. Sucrose fractions were collected and analyzed for mAzami and mCherry specific fluorescence (green and red bars). A254 profiles and fluorescence bar charts were superimposed and sucrose fractions were analyzed for presence of 16S and 23S rRNA by agarose gelelectrophoresis and subsequent optical inspection. Open circles: No rRNA, gray circles: fractions with low-intermediate amounts of rRNA, black circles: fractions with high amounts of rRNA.

## Abbreviations

mAzami green: A monomeric green fluorescent protein
mCherry: A monomeric red fluorescent protein
r-protein: ribosomal protein
r-RNA: ribosomal RNA
LB: Luria Broth
PCR: Polymerase chain reaction
SDS-PAGE: Sodium dodecyl sulphate polyacrylamide gel electrophoresis
IPTG: Isopropyl β-D-1-thiogalacto-pyranoside
UV: Ultraviolet
