# Supplementary material for: Fluorescence-based monitoring of ribosome assembly landscapes
Source: BMC Mol Biol. 2015 Feb 25;16:3. doi: 10.1186/s12867-015-0031-y (PMC4344731; doi:10.1186/s12867-015-0031-y)
Supplement: Additional file 1: — Overview constructed strains and 70S ribosome structure. (A) Surface representation of a T. termophilus 70S ribosome crystal structure. The 16S rRNA is colored in light gray proteins of the small subunit in yellow. 23S and 5S rRNA are shown in dark gray, proteins of the large subunit in cyan. S15 is highlighted in red, L1 in green. Their surface exposed C-termini are shown in purple. The figure was generated with pymol, based on PDB files 4KCZ and 4KCY (29). (B) Given are the names of the constructed strains as used in this study (lab nomenclature in brackets), relevant genotype and fluorescent fusion proteins produced. pTRC-rpsQ, pTRC-rplC: complementation plasmids with copies of the chromosomally deleted genes. Endogenous genes are shown as gray arrows, genes encoding fluorescent proteins as colored boxes. Genes to be deleted were replaced by kanamycin resistance cassettes (KanR). mCherry gene and protein portions are shown in red, mAzami accordingly in green. [file 12867_2015_31_MOESM1_ESM.pdf]

Additional File 1

A

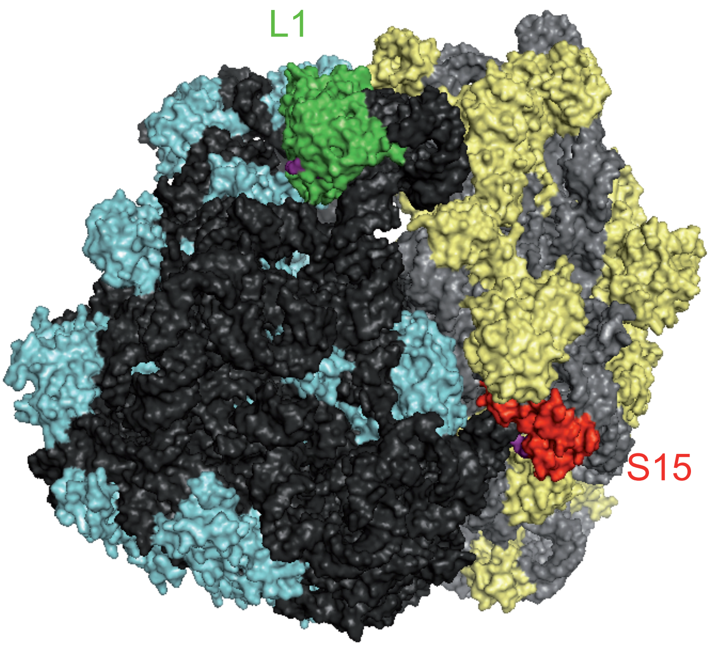

B

Strain	plasmid	relevant Genotype	Fusion proteins
**MCr*** (RN93.1)		*rpsO* -*mcherry*	S15 mCherry
**MCg*** (RN119.1)		*rplA* -*mazami*	L1 mAzami
**MCrg*** (RN115)		*rpsO* -*mcherry* *rplA* -*mazami*	S15 mCherry L1 mAzami
**MCΔsQ** (RN87)	*pTRC-rpsQ*	KanR ~~*rpsQ*~~	
**MCΔlC** (RN99)	*pTRC-rplC*	KanR ~~*rplC*~~	
**MCrg*ΔsQ** (RN116)	*pTRC-rpsQ*	*rpsO* -*mcherry* *rplA* -*mazami* KanR ~~*rpsQ*~~	S15 mCherry L1 mAzami
**MCrg*ΔlC** (RN117)	*pTRC-rplC*	*rpsO* -*mcherry* *rplA* -*mazami* KanR ~~*rplC*~~	S15 mCherry L1 mAzami

Additional File 1. Overview constructed strains and 70S ribosome structure.
(A) Surface representation of a T. termophilus 70S ribosome crystal structure. The 16S rRNA is colored in light gray proteins of the small subunit in yellow. 23S and 5S rRNA are shown in dark gray, proteins of the large subunit in cyan. S15 is highlighted in red, L1 in green. Their surface exposed C-termini are shown in purple. The figure was generated with pymol, based on PDB files 4KCZ and 4KCY (29).
(B) Given are the names of the constructed strains as used in this study (lab nomenclature in brackets), relevant genotype and fluorescent fusion proteins produced. pTRC-rpsQ, pTRC-rplC: complementation plasmids with copies of the chromosomally deleted genes. Endogenous genes are shown as gray arrows, genes encoding fluorescent proteins as colored boxes. Genes to be deleted were replaced by kanamycin resistance cassettes (KanR). mCherry gene and protein portions are shown in red, mAzami accordingly in green.
