# Supplementary material for: Fluorescence-based monitoring of ribosome assembly landscapes
Source: BMC Mol Biol. 2015 Feb 25;16:3. doi: 10.1186/s12867-015-0031-y (PMC4344731; doi:10.1186/s12867-015-0031-y)
Supplement: Additional file 2: — in vivo 30S and 50S ribosome assembly maps adapted from [ 10 ]. (A) 30S assembly map reflecting order and interdependency of r-protein interaction with the nascent small ribosomal subunit. Early interacting r-proteins are shaded in dark gray, late interacting ones in light gray. Boxed proteins are contained in the 21S precursor of the 30S subunit. S15 is circled in red. (B) 50S assembly map reflecting order and interdependency of r-protein interaction with the nascent large ribosomal subunit. Early interacting r-proteins are shaded in dark gray, late interacting ones in light gray. Proteins contained in the 32S or in the 43S precursor of the large ribosomal subunit are boxed or circled in black, respectively. L1 is circled in green. [file 12867_2015_31_MOESM2_ESM.pdf]

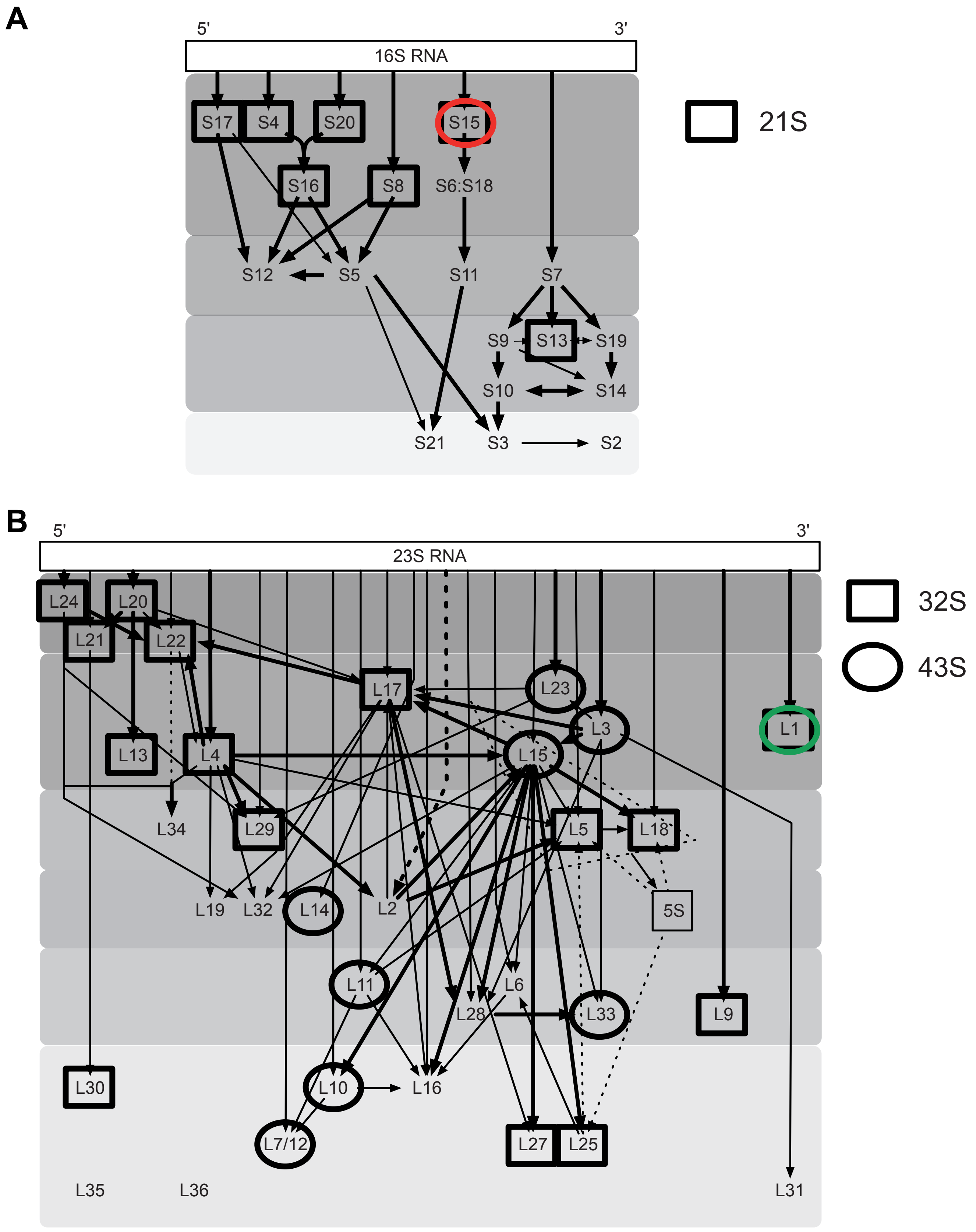

Additional File 2. in vivo 30S and 50S ribosome assembly maps adapted from [10].
(A) 30S assembly map reflecting order and interdependency of r-protein interaction with the nascent small ribosomal subunit. Early interacting r-proteins are shaded in dark gray, late interacting ones in light gray. Boxed proteins are contained in the 21S precursor of the 30S subunit. S15 is circled in red. (B) 50S assembly map reflecting order and interdependency of r-protein interaction with the nascent large ribosomal subunit. Early interacting r-proteins are shaded in dark gray, late interacting ones in light gray. Proteins contained in the 32S or in the 43S precursor of the large ribosomal subunit are boxed or circled in black, respectively. L1 is circled in green.
