# Supplementary material for: Fluorescence-based monitoring of ribosome assembly landscapes
Source: BMC Mol Biol. 2015 Feb 25;16:3. doi: 10.1186/s12867-015-0031-y (PMC4344731; doi:10.1186/s12867-015-0031-y)
Supplement: Additional file 3: — Alignment of mAzami specific fluorescence intensities from samples analyzed in Figure 4. mAzami fluorescence profiles from untreated cells in combination with mAzami profiles derived from (A) chloramphenicol (Cam), (B) erythromycin (Ery), (C) kanamycin (Kan) and (D) neomycin (Neo) treated cells. The diagrams show normalized mAzami fluorescence intensities from sucrose fractions derived from untreated cells (gray bars) in direct comparison with the ones from antibiotic treated cells (green bars). 70S peaks were used for normalization. [file 12867_2015_31_MOESM3_ESM.pdf]

Additional File 3

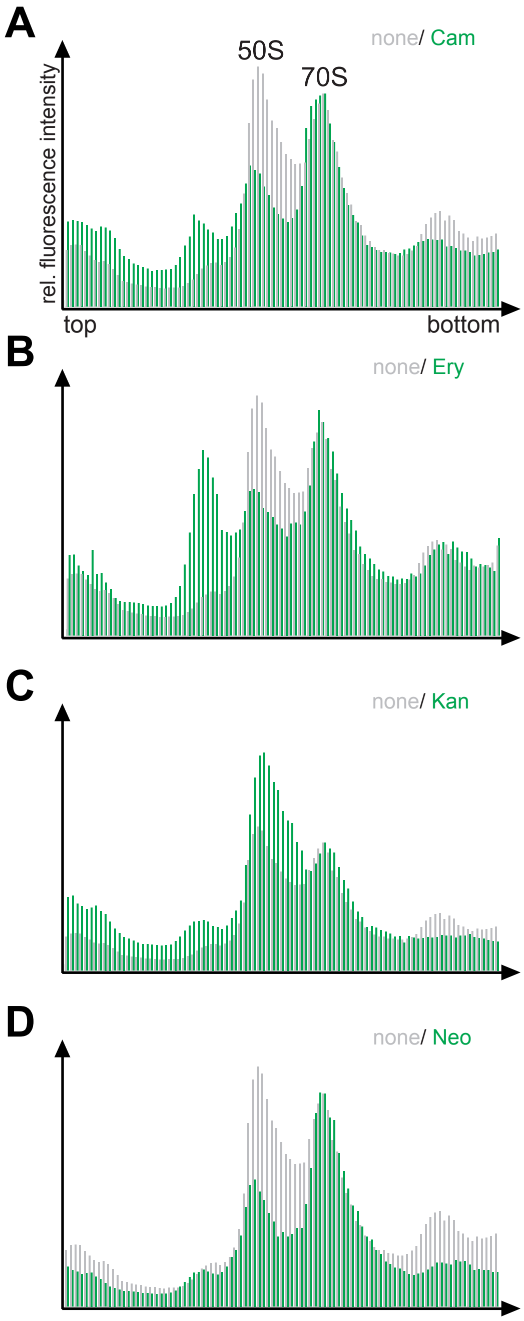

Additional File 3: Alignment of mAzami specific fluorescence intensities from samples analyzed in Figure 4
mAzami fluorescence profiles from untreated cells in combination with mAzami profiles derived from (A) chloramphenicol (Cam), (B) erythromycin (Ery), (C) kanamycin (Kan) and (D) neomycin (Neo) treated cells. The diagrams show normalized mAzami fluorescence intensities from sucrose fractions derived from untreated cells (gray bars) in direct comparison with the ones from antibiotic treated cells (green bars). 70S peaks were used for normalization.
