# Supplementary material for: Fluorescence-based monitoring of ribosome assembly landscapes
Source: BMC Mol Biol. 2015 Feb 25;16:3. doi: 10.1186/s12867-015-0031-y (PMC4344731; doi:10.1186/s12867-015-0031-y)
Supplement: Additional file 4: — Alignment of mCherry specific fluorescence intensities from samples analyzed in Figure 4. mCherry fluorescence profiles from untreated cells in combination with mCherry profiles derived from (A) chloramphenicol (Cam), (B) erythromycin (Ery), (C) kanamycin (Kan) and (D) neomycin (Neo) treated cells. The diagrams show normalized mCherry fluorescence intensities from sucrose fractions derived from untreated cells (gray bars) in direct comparison with the ones from antibiotic treated cells (red bars). 70S peaks were used for normalization. [file 12867_2015_31_MOESM4_ESM.pdf]

Additional File 4

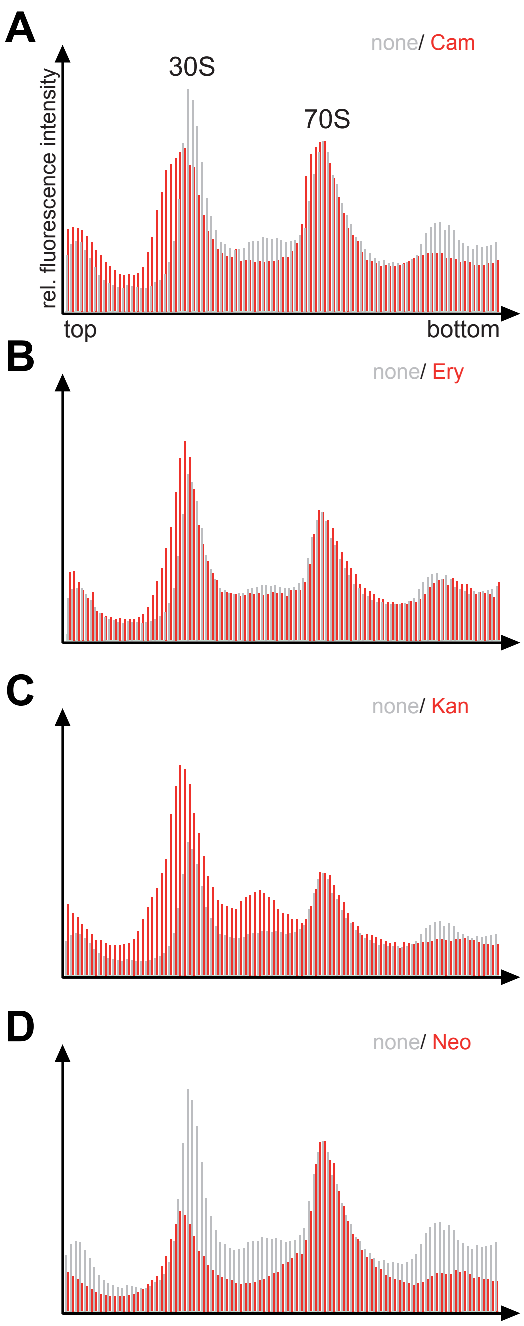

Additional File 4: Alignment of mCherry specific fluorescence intensities from samples analyzed in Figure 4
mCherry fluorescence profiles from untreated cells in combination with mCherry profiles derived from (A) chloramphenicol (Cam), (B) erythromycin (Ery), (C) kanamycin (Kan) and (D) neomycin (Neo) treated cells. The diagrams show normalized mCherry fluorescence intensities from sucrose fractions derived from non treated cells (gray bars) in direct comparison with the ones from antibiotic treated cells (red bars). 70S peaks were used for normalization.
