# Supplementary material for: Fluorescence-based monitoring of ribosome assembly landscapes
Source: BMC Mol Biol. 2015 Feb 25;16:3. doi: 10.1186/s12867-015-0031-y (PMC4344731; doi:10.1186/s12867-015-0031-y)
Supplement: Additional file 5: — A254 detection and fluorescence analysis of sucrose density gradient fractions. Sucrose density gradient (10-25%) centrifugation profiles derived from (A) control cells with no antibiotic (none), (B) chloramphenicol (Cam), (C) erythromycin (Ery), (D) kanamycin (Kan) and (E) neomycin (Neo) treated cells. Sucrose fractions were collected and analyzed for mAzami and mCherry specific fluorescence (green and red bars). A254 profiles and fluorescence bar charts were superimposed and sucrose fractions were analyzed for presence of 16S and 23S rRNA by agarose gelelectrophoresis and subsequent optical inspection. Open circles: No rRNA, gray circles: fractions with low-intermediate amounts of rRNA, black circles: fractions with high amounts of rRNA. [file 12867_2015_31_MOESM5_ESM.pdf]

Additional File 5

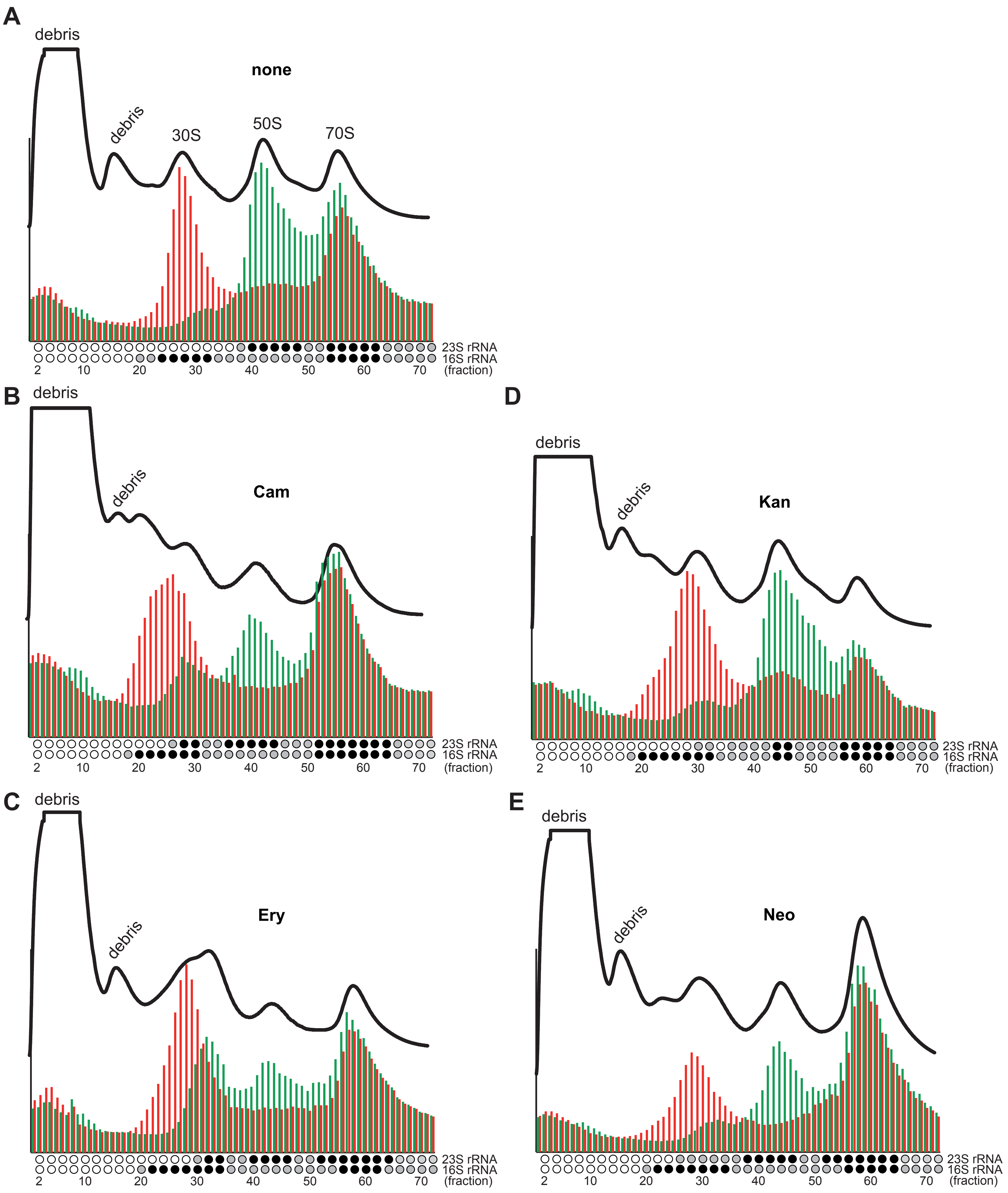

Additional File 5: A254 detection and fluorescence analysis of sucrose density gradient fractions
Sucrose density gradient (10-25%) centrifugation profiles derived from (A) control cells with no antibiotic (none), (B) chloramphenicol (Cam), (C) erythromycin (Ery), (D) kanamycin (Kan) and (E) neomycin (Neo) treated cells. Sucrose fractions were collected and analyzed for mAzami and mCherry specific fluorescence (green and red bars). A254 profiles and fluorescence bar charts were superimposed and sucrose fractions were analyzed for presence of 16S and 23S rRNA by agarose gelelectrophoresis and subsequent optical inspection. Open cirlces: No rRNA, gray circles: fractions with low-intermediate amounts of rRNA, black circles: fractions with high amounts of rRNA.
